# Trans-Polyisoprene/Poly (Ethylene-co-Vinyl Acetate) Polymer Composites as High-Performance Triple Shape Memory Materials

**DOI:** 10.3390/polym14245344

**Published:** 2022-12-07

**Authors:** Hua Xin, Yangfan Li, Qi Peng

**Affiliations:** 1 College of Chemistry and Chemical Engineering, Shaanxi University of Science and Technology, Xi’an 710021, China; 2 Key Laboratory of Chemical Additives for China National Light Industry, Shaanxi University of Science and Technology, Xi’an 710021, China; 3 Xi’an Key Laboratory of Advanced Performance Materials and Polymers, Shaanxi University of Science and Technology, Xi’an 710021, China

**Keywords:** shape memory effect, trans-polyisoprene, poly (ethylene-co-vinyl acetate), crystallization, compatibility

## Abstract

The performance and programming conditions of the triple shape memory of crosslinked trans-polyisoprene/poly (ethylene-co-vinyl acetate) (TPI/EVA) composites with different contents of dicumyl peroxide (DCP) were investigated. The effect of triple shape memory in the TPI/EVA composites was studied by tensile loading, scanning electron microscopy (SEM), differential scanning calorimetry (DSC), and dynamic thermomechanical analysis (DMA). It was demonstrated that the content of DCP increased, the crystallization temperature of TPI decreased from 55.2 to 38.3 °C, and the crystallization temperature of EVA decreased slightly. The SEM results showed that DCP, as an initiator, could form a graft copolymer of TPI-g-EVA at the interface of the two phases, which could improve the adhesion of the two phases. The DMA showed that the higher the content of DCP, the higher the first-stage shape recovery ratio. Moreover, the composites exhibited favorable shape fixity ratio (R_f_) and shape recovery ratio (R_r_) with the incorporation of 0.4 phr DCP. At the same time, it was demonstrated that the TPI/EVA composites showed excellent mechanical strength, including tensile strength up to 24.3 MPa, as well as elongation at break reaching 508%.

## 1. Introduction

Ordinary shape memory polymers (SMPs) typically consist of a stationary phase and reversible phase. The stationary phase is usually made up of chemical or physical crosslinking points used to memorize the original shape, and the reversible phase can be used to fix the temporary shape [1]. In most of the previous research [2,3,4,5], the study of shape memory polymers has been largely focused on multiple reversible phase transitions [6]. There are two techniques to achieve this effect, which are chemical synthesis and physical blending. Through physical blending, polymer composites can have different reversible phases or unique stationary phase designs. Compared with the chemical synthesis method, the production cost of polymer blending is lower, its operation is more straightforward, and it is more suitable for mass production.

Trans-polyisoprene (TPI) is a natural polymer material with rubber and plastic properties, and it has a wide range of applications. TPI, as a colloidal component, exists in abundance in the leaves, skins, and other parts of *eucommia ulmoides*. TPI has been widely used as a shape memory material in previous studies because it is crystalline under room temperature conditions and amorphous above its melting temperature [7,8,9,10]. However, TPI is mixed with other polymer materials when it is used to design triple shape memory polymers. Ming Tian et al. reported a new type of triple shape memory elastomer by applying paraffin in the reversible phase, and the addition of low crosslinked TPI fixed the second temporary shape and provided a driving force for shape recovery [11]. Yan Wang et al. prepared TPI/high-density polyethylene (HDPE) triple shape memory polymer composites with a tensile strength of 15 MPa and a tear strength of 51 KN/m^2^ [10].

However, there have been relatively few studies on the compatibility of triple SMPs based on TPI and other materials. Poly (ethylene-vinyl acetate) (EVA) is considered to be an essential material used for SMPs because of its good low-temperature resistance, processability, and mechanical properties [12,13,14]. The chain segment of EVA consists of two different parts, ethylene segments and vinyl acetate segments. The crystalline vinyl segments can be expected to be compatible with TPI, and the amorphous vinyl acetate segments give the EVA its elasticity. As a result, as a general-purpose polymer, EVA is fabricated for use as a shape memory material through thermal or radiation crosslinking [15,16,17]. Therefore, the DCP can be used as a crosslinking agent for EVA. The mechanism is as follows: the peroxy bond breaks in DCP to form a peroxy radical RO, which is quickly bound to the H of the alkyl group on the EVA branch chain, and the two alkyl active groups combine to form a crosslinked EVA. In addition, TPI can also be crosslinked by DCP [18]. Based on the mechanism of DCP crosslinking, the segments of TPI and EVA were connected. Therefore, DCP can increase compatibility through a free-radical-initiated crosslinking reaction. Longyi Sun et al. fabricated ethylene-acrylic acid graft copolymer (EAA) and ethylene-vinyl acetate graft copolymer blends with DCP as the crosslinking agent; the first shape fixity ratio was about 90%, and the second shape fixity ratio was about 90% [19]. Xin Zhou et al. synthesized a green poly (butylene succinate) (PBS) lignin gel compatibilizer with a new network structure through the free radical grafting of PBS and lignin initiated by DCP during melt extrusion [20].

In this work, the interfacial compatibilization reaction between TPI and EVA induced by DCP and the effects of different mass fractions of DCP on the mechanical properties, crystalline properties, interface morphology, and triple shape memory of composites were studied. This research attempts to adjust compatibility in a TPI/EVA polymer composite to adapt the triple shape memory properties of polymer materials. The prepared composites exhibited excellent triple shape memory effects and mechanical properties, which can possibly be utilized in the fields of robotics, actuators, and biomedicine. This work will help to design triple shape memory polymers with good compatibility and provides a basis for the practical application of traditional commercial polymers.

## 2. Materials and Methods

### 2.1. Materials

Trans-polyisoprene (TPI) was obtained from Qingdao Dipai New Material Co., Ltd., Qingdao, China. Poly (ethylene-co-vinyl acetate) (EVA) with 18 wt% vinyl acetate was purchased from Jiangsu Sierbang Petrochemical CO., LTD, Nanjing, China; it’s melting temperature is about 90 °C. Dicumyl peroxide (DCP) was provided by Tianjin HongYan Reagents, Tianjin, China. Stearic acid (SA) and zinc oxide (ZnO) were supplied from Yongda Chemistry, Tianjin, China.

### 2.2. Samples Preparation

In the experiments, TPI and EVA were dried in an oven at 60 °C before use. Firstly, TPI was mixed in two roll mills at 70 °C, and SA, ZnO, EVA, and DCP were added in turn. Then, the samples were vulcanized on a flat vulcanizing machine under 175 °C × vulcanization (T_90_) time and a pressure of 10 MPa to obtain a crosslinked composite with 2 mm thickness. In this study, the mass ratio of TPI and EVA was fixed at 7:3, and the obtained crosslinked composites were named T/E-Dx, where x reflects the weight percentage of DCP.

### 2.3. Characterization

#### 2.3.1. Vulcanization Characteristic Curve

The vulcanization characteristic refers to the curve points that determine the relationship between the physical properties of rubber in the vulcanization process and the vulcanization time at a specific temperature. The cured properties of the TPI/EVA composites were measured with a UR-2010 Rheometer (U-CAN Dynates Inc, Taiwan, China)

#### 2.3.2. Equilibrium Swelling Experiment

A sample with a mass of m_1_ was chopped into 1 mm pieces and immersed in toluene for 7 d. The mass of the swollen sample was marked as m_2_, and the crosslink density (*Vr*) of the sample after toluene extraction was calculated according to Equation (1):

(1) $$Vr=\frac{1}{1+\left(m_{2}+m_{1}-1\right)\times \rho_{r}/\alpha \rho_{s}}\times 100\%,$$

where *ρ_r_* and *ρ_s_* are TPI and toluene density (*ρ_r_* = 0.980 g/cm^3^, *ρ_s_* = 0.866 g/cm^3^), respectively, and *α* is the mass ratio of the EVA in the composites.

#### 2.3.3. Tensile Testing

A universal testing machine was used to carry out the tensile test on dumbbell-shaped samples. The tensile speed was kept at 500 mm/min.

#### 2.3.4. X-ray Diffractometer

X-ray diffraction (SmartLab 9Kw, Rigaku Corporation, Akishima-shi, Japan) was used to characterize the crystal structure of polymer composites. The diffraction angle was 5–80°, and the scanning rate was 5° min^−1^.

#### 2.3.5. Scanning Electron Microscopy (SEM)

The morphology of the samples was recorded with a FEI Q45 + EDAX Octane Prime (FEI and EDAX Company, Hillsboro, OR, USA). In order to observe the phase structure of TPI and EVA, the samples need to be quenched cryogenically and sprayed with gold on the section.

#### 2.3.6. Differential Scanning Calorimetry (DSC)

The crystallization and melting characteristics of TPI and EVA were measured in polymer materials by a DSC (Q2000, TA Instruments, New Castle, PA, USA) at 10 °C min^−1^. After keeping the sample at 120 °C for 5 min, it was cooled down to −10 °C and then elevated to 120 °C.

#### 2.3.7. Fourier Transform Infrared Spectra (FT-IR)

Fourier transform infrared (FT-IR) spectra were tested with a Bruker VECTOR-22 equipped with an ATR accessory. The wavenumbers ranged from 4000 to 400 cm^−1^ for 32 scans. In addition, the samples were immersed in toluene for 7 d to remove the free EVA from the blends; then, the samples were dried for 24 h at 80 °C.

#### 2.3.8. Triple Shape Memory Characterization 

The triple shape memory behavior was characterized by controlled force mode in dynamic thermomechanical analysis (DMA TQ800) at a 1 Hz frequency. The shape memory test steps were as follows: For the first step, the TPI/EVA sample with a size of 30 mm × 6 mm × 2 mm was heated at 105 °C for 5 min, and the strain was denoted as S_0_. The second step was to fix the first temporary shape. In addition, the sample was stretched under a tension of 0.10 MPa, and the temperature dropped below the crystallization temperature (Tc) of the EVA, but above the Tm of the TPI. The strain was denoted at this point as *S*_1*,load*_. After an isothermal period of 2.00 min, the stress was removed, and the strain was recorded as S_1_. For the third step, the second temporary shape was fixed. The TPI/EVA sample was extended at 55 °C with a stress of 0.75 MPa. While the stress was unchanged, the temperature of the sample dropped below the Tc of the TPI, and the sample equilibrated at this temperature for 20 min. The strain at this point was denoted as *S*_2*,load*_. Then, the stress was removed, and the strain was denoted as S_2._ The fourth step is for the sample to return to its first temporary shape. The sample was heated to 55 °C and then equilibrated for 10 min; the strain at this point was denoted as *S*_1*,rec*_. The final step was for the sample to return to its initial shape. The sample was heated to 105 °C for 10 min, and the strain at this point was denoted as *S*_2*,rec*_. Equations (2) and (3) were used to calculate the fixity ratio (*R_f_*) and recovery ratio (*R_r_*):

(2) $$R_{f}\left(X\to Y\right)=\frac{S_{Y}-S_{X}}{S_{\left(Y,load\right)}-S_{X}}\times 100\%,$$

(3) $$R_{r}\left(Y\to X\right)=\frac{S_{Y}-S_{\left(X,rec\right)}}{S_{Y}-S_{X}}\times 100\%,$$

where *S* and *S_load_* represent the strain after deformation and removal, respectively. *S_rec_* represents the recovered strain. A different thermodynamic state corresponds to the two variables *X* and *Y.* In addition, a digital camera recorded the triple shape memory performance of the composites.

## 3. Results

### 3.1. Vulcanization Characteristic Parameters

**Scheme 1:**
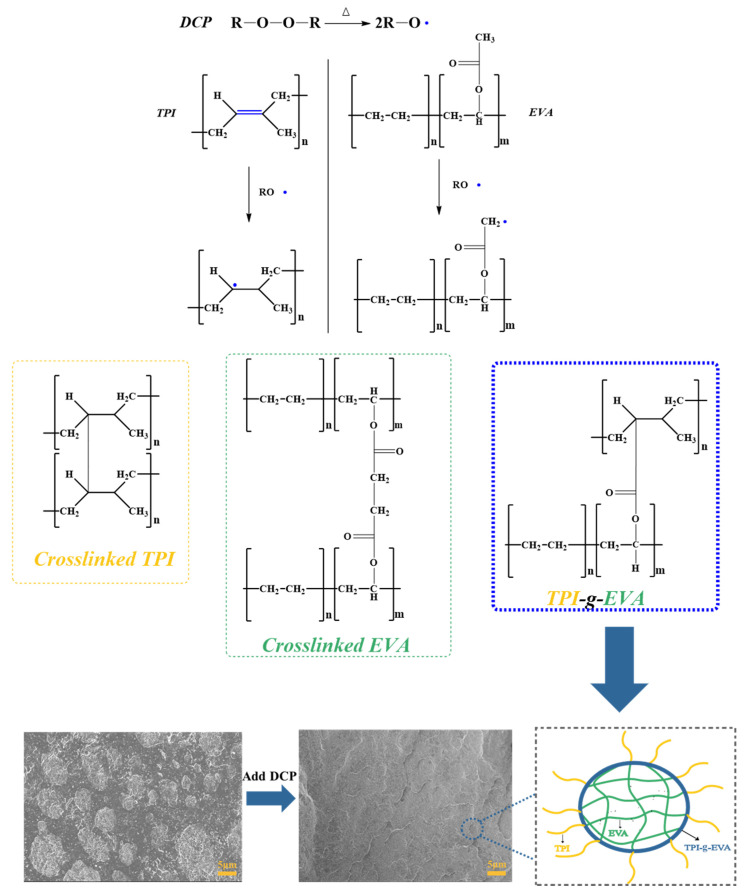
Reactive compatibilization of TPI/EVA composites after addition of DCP.

**Figure 1:**
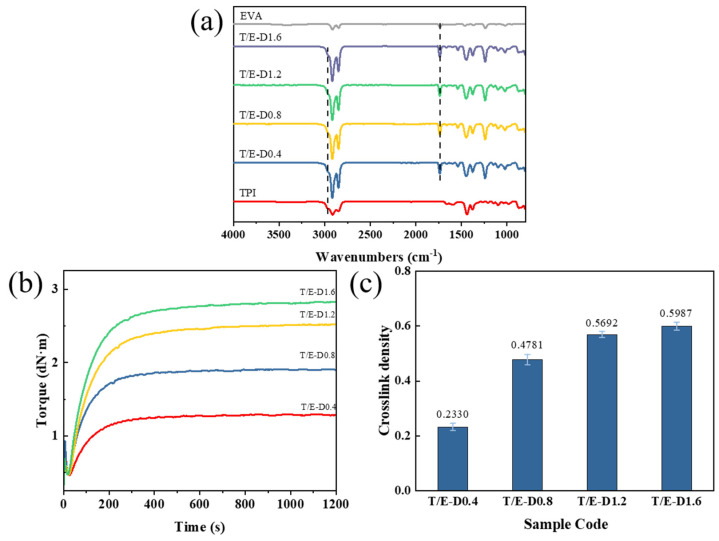
(**a**) FT-IR spectra of the toluene-extracted TPI/EVA composites; (**b**) Elastic torque of TPI/EVA with different contents of DCP at 175 °C, and (**c**) Column plots of crosslink density of toluene-extracted TPI/EVA composites with different contents of DCP.

**Figure 2:**
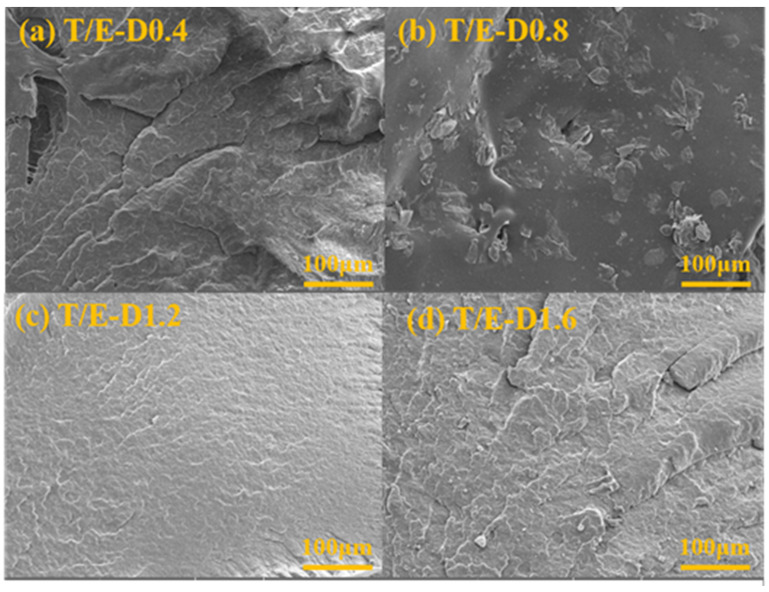
SEM micrograph of the cryogenically fractured surface of TPI/EVA composites with different DCP contents: (**a**) T/E-D0.4; (**b**) T/E-D0.8; (**c**) T/E-D1.2; (**d**) T/E-D1.6.

The compatibilization reaction mechanism diagram of the composite after adding dicumyl peroxide (DCP) is shown in Scheme 1. When the DCP was not added, the phases of TPI and EVA in the composites had an obvious interface due to their poor compatibility. With the addition of DCP, the free radicals were generated by the decomposition of DCP during the vulcanization process, and the free radicals initiated the simultaneous generation of free radicals from the Trans-polyisoprene (TPI) and Poly (ethylene-co-vinyl acetate) (EVA) segments. When the two free radicals came into contact, crosslinked TPI, TPI-g-EVA, and crosslinked EVA were formed [21,22]. To further confirm the occurrence of the chemical reaction, the TPI/EVA composites were extracted in toluene for 7 days to remove the unreacted EVA, and the spectra are shown in Figure 1a. The absorption peak at 1722 cm^−1^ was attributed to the ester group of the EVA, and that at 1975 cm^−1^ was attributed to the C=CH stretching vibration of the TPI. The TPI-g-EVA was distributed at the interface of TPI, and EVA acted as a compatibilizer, which improved the interfacial adhesion between the two phases (Figure 2).

The curing characteristics curve of TPI/EVA is shown in Figure 1b. The maximum torque, minimum torque, and vulcanization time are represented by M_H_, M_L,_ and T_90_, respectively. Stock modules and crosslink density were measured based on these values. To evaluate the crosslinking density of composites, torque disparity (M_H_-M_L_) can be used. As shown in Table 1, as a result of the addition of DCP content, M_H_-M_L_ continuously increased, from 0.83 to 2.37 dN·m, and the cure rate index of the composites also increased from 12.54 to 23.35 min^−1^, which indicated an increase in the crosslinking degree. In addition, as the DCP content increased, T_90_ gradually decreased, which meant that the increase in the concentration of DCP in the composites results in a shortening of T_90_. The TPI/EVA composites with different DCP contents were extracted with toluene to remove unreacted EVA, and the crosslink density is shown in Figure 1c. As the DCP content increased, the crosslinking density of the composites increased. This was consistent with the results of the cure characteristics curves.

### 3.2. Phase Morphology

**Figure 3:**
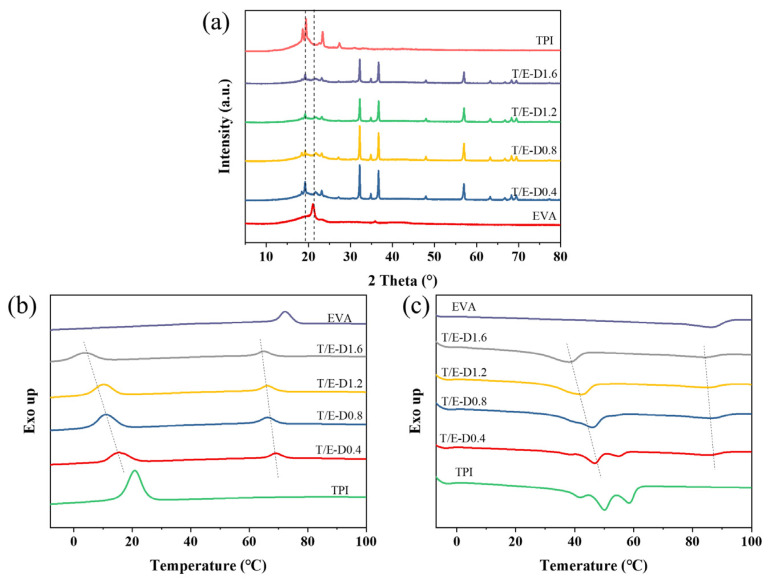
(**a**) X-ray diffractogram curves, (**b**) DSC cooling curves, and (**c**) secondary heating curves of the TPI/EVA composites.

The SEM micrograph of the cryo-fractured surface of the TPI/EVA composites is represented in Figure 2. It can be seen that the graph is rough and there are more gullies on the interface of the two phases in Figure 2a. As the content of DCP increased, the cryo-fractured interface of TPI/EVA composites gradually became smoother. As a result of increasing the content range of DCP from 0.4 to 1.2 phr, the interfacial compatibility was improved. Therefore, the samples exhibited a smooth fracture surface, which can be observed in Figure 1b,c. This phenomenon is due to the fact that DCP acted as an initiator to form the graft copolymer of TPI-g-EVA at the interface of the two phases, which improved the adhesion of the two phases (Scheme 1). With the increase in DCP content, the content of the graft copolymer increased, resulting in a gradually smoothing phase interface. However, phase separation is observed when the DCP content reached 1.6 phr in Figure 1d. The reason is that when the DCP content was 1.6 phr, the TPI crosslinking density increased, causing a significant reduction in the crystallization region. In other words, the high crosslinking density caused the Tc and Tm to become lower than room temperature. At this time, the TPI phase was elastic at room temperature, and the EVA phase was plastic, reducing two-phase separation as shown in Figure 3b,c.

### 3.3. Crystal Properties

The triple shape memory property of the TPI/EVA composite was based on the crystalline phases of TPI and EVA; therefore, the crystallization property of the composites was necessary to study. The XRD curves are as shown in Figure 3a, and the crystallized peaks of pure TPI and EVA were 2θ = 23.34° and 2θ = 21.68°, respectively. However, some new peaks appeared in the polymer composite curves: these were confirmed as ZnO peaks by standard XRD patterns (2θ = 37.76°, 34.42°, 36.25°, 47.54°, 53.59°). Moreover, in addition to the crystallization peaks of TPI, EVA, and ZnO in the XRD curves, there was no new crystallized peak visible, indicating that TPI and EVA crystallized independently: there was no co-crystallization occurring between the two phases [11]. In other words, the absence of new crystallized peaks meant the crystalline regions between reversible phases did not affect each other, which was necessary for triple shape memory performance.

The Tc and Tm of the polymer composite were typically the switching temperatures used in shape memory composites. Therefore, DSC measurements were conducted to test the Tc and Tm of the TPI and EVA phase. The DSC curves are shown in Figure 3b,c. The pure TPI demonstrated double melting peaks that are attributed to two Tms (β (50.1 °C) and α (58.5 °C)) and one Tc of 20.8 °C, and the EVA exhibited one melting peak of about 90.0 °C and one Tc of 72.3 °C, respectively. Although the ΔHm of EVA and TPI decreased, the temperature difference was greater than 20 °C, which confirmed that the crystallization temperature and melting temperature of the TPI and EVA were independent of each other, and the composite materials could be used to design triple shape memory materials. After the TPI and EVA were mixed, the TPI and EVA phases were connected by vulcanization. The Tc and Tm of the TPI and EVA phases decreased as the DCP content increased (Table 2). As a result, by adjusting the content of DCP, the temperature range used for triple shape memory composites can be controlled. However, more crosslinking points would destroy the crystalline region of TPI, which would be harmful to the first temporary shape fixing. The Tm/Tc of the EVA phase decreased slightly as the DCP content increased, while the TPI decreased rapidly, which demonstrated the DCP tended to react with the TPI double bonds. In addition, the DCP tended to destroy the α crystal phase of TPI. As a result, the peak of the α crystal phase disappeared when the DCP content increased.

### 3.4. Triple Shape Memory Property

**Figure 4:**
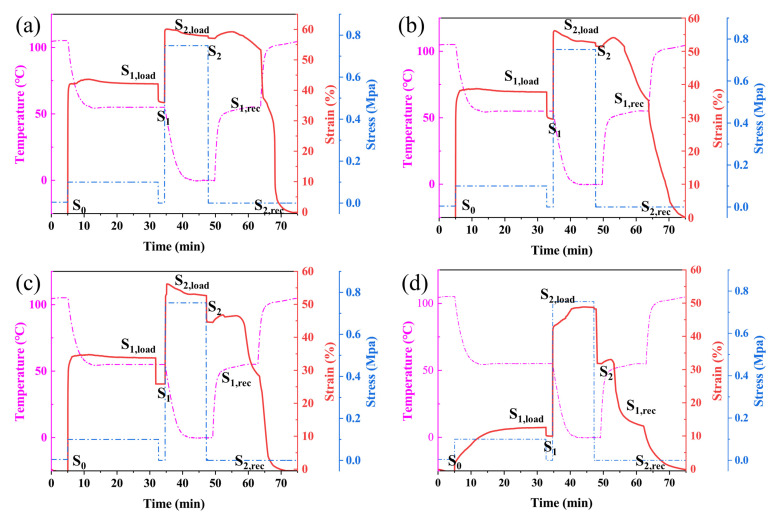
DMA curves of the triple memory shape memory behavior of the TPI/EVA composites: (**a**) T/P-D0.4; (**b**) T/P-D0.8; (**c**) T/P-D1.2; (**d**) T/P-D1.6.

The triple shape memory performance of the TPI/EVA composites was further researched using DMA. As shown in Figure 4, the red solid line represents the strain, the blue dotted line represents the applied stress, and the pink dotted line represents the temperature. According to the DSC results above, at 105 °C, the TPI and EVA phases were in a melting state. The sample was not stressed at this time, and the initial strain of 0% was denoted as S_0_. Then, a stress of 0.10 MPa was applied, and the sample was easily stretched to *S*_1*,load*_. After reducing the temperature to 55 °C and removing the stress, the crystalline region of TPI was melting, the EVA was in a crystalline state, and the strain of the sample shrank to S_1_. Then, a stress of 0.75MPa was applied, and the sample was stretched to *S*_2*,load*_. Subsequently, after cooling the sample rapidly to 0 °C and removing the stress of 0.75 MPa, the deformation of the sample was denoted as S_2_, and all phases were in a crystalline state. Then, after being heated up to 55 °C, the crystalline region of TPI was transformed into an amorphous state, and due to the existence of the crosslinked network, shape deformation occurred and the TPI was restored to the first temporary shape. The strain of sample was restored to *S*_2*,rec*_. Finally, when the temperature reached 105 °C, the sample was converted to *S*_1*,rec*_ because the crystalline region of EVA was in a melting state.

The shape fixity ratio R_f_ and the shape recovery ratio R_r_ of samples with differing DCP contents were examined. As shown in Table 3, in order to compute R_f_ and R_r_, formulae 1 and 2 were used. At 55 °C, *R_f_* (0→1) (origin shape → shape 1) was the first temporary shape ratio, which was controlled by the crystallization of the EVA phase. As the peroxides tended to react with the TPI, the damage to the EVA crystallization regions was less severe, resulting in *R_f_* (0→1) increasing with the DCP content. At 0 °C, the fixed ratio of the second temporary shape memory was denoted as *R_f_* (1→2) (shape 1→shape 2), and the magnitude of its value depended mainly on the crystallization regions of the TPI. The peroxides tended to react with the segments of the TPI, increasing the crosslinking density, and destroying the crystallization regions, which had been demonstrated by DSC and SEM. Therefore, the value of *R_f_* (1→2) dramatically decreased as the DCP content increased. Moreover, although *R_f_* (1→2) decreased, the recovery speed increased. The driving force for shape recovery came from the density of the crosslinking network: the higher the density of the crosslinking network, the higher the shape recovery ratio. During the shape recovery process of the first stage, when heated from 0 to 55 °C, the crystallization phase of the TPI was melting, while the EVA was still in the crystallization state. The recovery ratio *R_r_* (2→1) (shape 2→shape 1) and recovery ratio R_r_ (1→0) (shape 1 → origin shape) were gradually increased.

**Figure 5:**
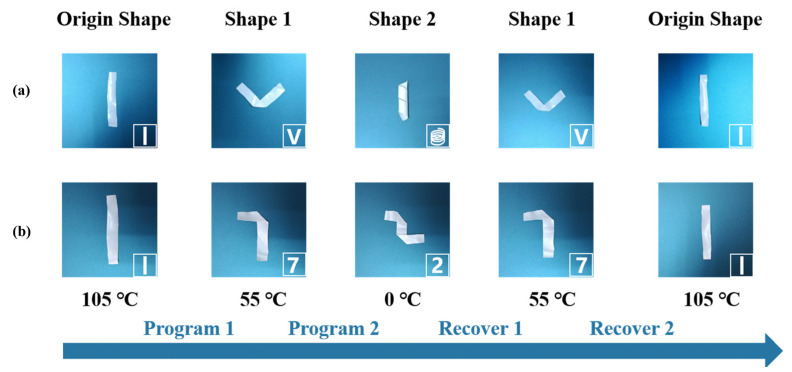
Photographs of the triple shape memory effect of the T/E-D0.4 composite: (**a**) first shape memory cycle; (**b**) second shape memory cycle.

Digital photographs were used to determine the macro-level triple shape memory performance. As shown in Figure 5, a folded flat rectangular sample at 105 °C was reshaped at 55 °C to form a “V” shape. Then, the “V”-shaped sample was transformed into a spiral shape at 0 °C. Under heating conditions, the spiral shape returned to a “V” shape at 55 °C and a rectangle at 105 °C. The sample was fixed to two shapes during the process of applying external force, and it recovered its origin shape. To further illustrate that the triple shape memory behavior of the sample could be repeated, another shape memory cycle is demonstrated in Figure 5b. Similarly, under the same circumstances, the sample deformed to a “7” shape at 55 °C and fixed to a “2” shape at 0 °C. Then, the sample was converted into the origin shape again. It was proven that the TPI/EVA composites had excellent triple shape memory behavior, which broadens the the industrialized applications of shape memory materials.

### 3.5. Mechanical Property

**Figure 6:**
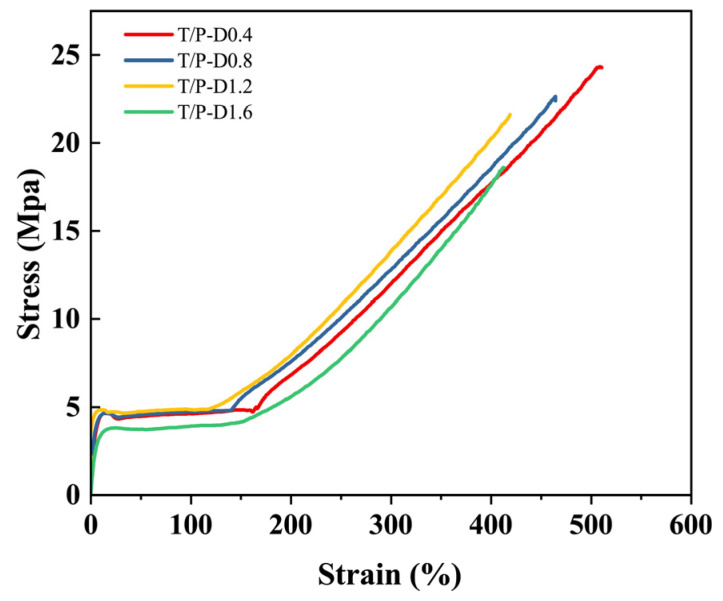
Stress–strain curves of T/E-Dx composites.

The mechanical property determined the application range of shape memory composites. Tensile strength was affected by the crosslinking density and crystallization in the polymer composites. The tensile strength of the composites was mainly provided by the crystalline properties of the TPI. The crosslinking density produced by DCP destroyed the crystalline regions of TPI/EVA polymer composites, resulting in a gradual decrease in the mechanical properties with the increase in the DCP content. As shown in Figure 6, when the DCP content was 0.4 phr, the tensile strength reached 24.3 MPa, and its elongation at break reached 508%. As the DCP content increased, the mechanical strength and elongation at the break of the composites decreased. When the DCP content was 1.6 phr, the tensile strength reached 18.6 MPa, and its elongation at break reached 412%.

### 3.6. Shape Memory Mechanism

**Scheme 2:**
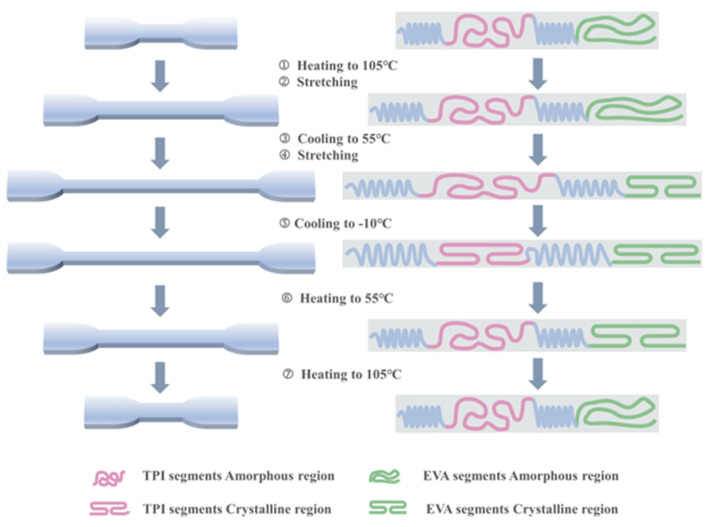
Schematic diagram of TPI/EVA composites in triple shape memory process.

The mechanism of triple shape memory in TPI/EVA composites is shown in Scheme 2. to explain the process of shape memory storage and release. The phase structure of the composites was essential for shape memory behavior. For compatible polymer composites, the two components can be regarded as a continuous mechanism through a chemical bond connection between phases, and different shapes can be controlled through different crystallization regions. While the shape was restored, the internal stress could be transmitted through a TPI-g-EVA graft copolymer. As shown in Scheme 2, the red disordered and ordered patterns represent the amorphous and crystallization regions of the TPI, respectively; the corresponding green patterns represent EVA segments.

At room temperature, there were three regions in the TPI and EVA composites: the crystallization, crosslinking, and amorphous region, respectively. The crystallization region controlled the fixed shape, and the crosslinking density determined the shape recovery process and speed. At 105 °C, the crystalline regions in the composite were all melted down, and the sample was easily stretched (steps 1–2). When cooled to 55 °C and maintaining stress, the green EVA segments were regularly stacked under force and temperature to form a regular crystalline region (steps 3–4). Then, when cooled to 0 °C, the segments of the TPI were arranged under induced force and temperature increases to form a red crystalline region (step 5). When heated up to 55 °C, the TPI crystalline region began melting and releasing the stored internal stress (step 6). At 105 °C, the EVA crystal region began melting and releasing internal stress, and the sample returned to its initial shape (step 7).

## 4. Conclusions

This work focused on the triple shape memory effect of DCP content as a compatibilizer through the crosslinking reactions of TPI/EVA composites. It was found that the graft copolymer TPI-g-EVA was formed by a DCP-induced free radical reaction. The polymer composites showed a smooth structure at high DCP content, and there were two crystallization and melting peaks. By increasing DCP content, the Tc and Tm of TPI/EVA polymer composites gradually decreased, which was attributed to the degree of crosslinking density. Crosslinking density increased as the DCP content increased, inhibiting molecular movement and hindering crystallization. However, the *R_f_* (1–2) of T/E-D1.6 decreased from 88.2% to 57.4%, indicating that they had difficulty maintaining the second temporary shape. The shape memory properties of composites with DCP contents of 0.4 phr were satisfactory, with a second shape fixed ratio of 88.2% and a first shape recovery ratio of 71.1%. In terms of mechanical properties, the T/E-D0.4 was capable of tensile strengths up to 24.3 MPa and elongation at break up to 508%.

## Figures and Tables

**Table 1:** Result of the vulcanization characteristic curve.

Properties\Sample Code	T/E-D0.4	T/E-D0.8	T/E-D1.2	T/E-D1.6
M_H_ (dN·m)	1.30 ± 0.13	1.91 ± 0.09	2.53 ± 0.16	2.84 ± 0.11
M_L_ (dN·m)	0.47 ± 0.06	0.48 ± 0.02	0.47 ± 0.05	0.47 ± 0.03
M_H_-M_L_ (dN·m)	0.83 ± 0.19	1.43 ± 0.11	2.06 ± 0.21	2.37 ± 0.14
T_10_ (min)	0:41 ± 0:02	0:35 ± 0:02	0:39 ± 0:02	0:34 ± 0:01
T_90_ (min)	8:40 ± 1:22	5:17 ± 0:36	5:04 ± 0:12	4:51 ± 0:22
Cure rate index (min^−1^)	12.54 ± 2.56	21.34 ± 1.44	22.63 ± 1.83	23.35 ± 0.87

M_H_ represents the shear modulus, hardness, tensile strength, and crosslinking density of rubber. M_L_ represents the fluidity of rubber. T_10_ represents the safe operation time of rubber. T_90_ was mainly used to evaluate the shortest molding time of rubber in production. Cure rate index reflects the speed of vulcanization reaction.

**Table 2:** Tc, Tm of TPI/EVA composites with different DCP contents.

Properties\Sample Code	T/E-D0.4	T/E-D0.8	T/E-D1.2	T/E-D1.6
T_c,TPI_ (°C)	15.3 ± 2.3	10.9 ± 1.8	10.1 ± 1.3	3.33 ± 2.2
T_c,EVA_ (°C)	68.8 ± 3.2	66.1 ± 2.1	65.9 ± 3.1	64.7 ± 1.0
ΔH_m(TPI)_ (J/g)	19.1 ± 2.3	13.7 ± 3.0	11.9 ± 1.1	6.73 ± 2.9
T_m,TPI_ (°C)	55.2 ± 2.2(α) 47.0 ± 2.3(β)	46.2 ± 3.2	42.8 ± 3.3	38.3 ± 1.0
T_m,EVA_ (°C)	87.0 ± 0.6	86.0 ± 0.3	85.7 ± 0.4	84.7 ± 0.3
ΔH_m(EVA)_ (J/g)	7.77 ± 0.8	6.47 ± 0.2	5.23 ± 0.4	4.37 ± 0.3

**Table 3:** Triple shape memory performance of the TPI/EVA composites.

Properties\Sample Code	T/E-D0.4	T/E-D0.8	T/E-D1.2	T/E-D1.6
R_f_ (0→1)(%)	85.3 ± 3.2%	88.2 ± 1.2%	71.1 ± 5.5%	99.6 ± 0.2%
R_f_ (1→2)(%)	79.9 ± 2.4%	82.2 ± 0.8%	77.3 ± 4.6%	99.7 ± 0.1%
R_r_ (2→1)(%)	79.8 ± 2.0%	67.6 ± 0.8%	78.9 ± 4.3%	99.9 ± 0.1%
R_r_ (1→0)(%)	79.4 ± 1.9%	57.4 ± 0.9%	81.8 ± 2.1%	99.8 ± 0.1%

## Data Availability

Not applicable.

## References

[1] Chen: C.-M., Chiang C.-L., Lai C.-L., Xie T., Yang S. (2013). Buckling-Based Strong Dry Adhesives Via Interlocking. Adv. Funct. Mater..

[2] Karasu F., Weder C. (2020). Blends of poly(ester urethane)s and polyesters as a general design approach for triple-shape memory polymers. J. Appl. Polym..

[3] Yang K., Du J., Zhang Z., Ren T. (2019). A facile strategy to fabricate robust triple-shape memory polymer. Mater. Lett..

[4] Amini M., Wu S. (2021). Designing a polymer blend nanocomposite with triple shape memory effects. Compos. Commun..

[5] Wang Y., Pan Y., Zheng Z., Ding X. (2018). Reconfigurable and Reprocessable Thermoset Shape Memory Polymer with Synergetic Triple Dynamic Covalent Bonds. Macromol. Rapid Commun..

[6] Melly S.K., Liu L., Liu Y., Leng J. (2020). Active composites based on shape memory polymers: Overview, fabrication methods, applications, and future prospects. J. Mater. Sci..

[7] Kang H., Gong M., Xu M., Wang H., Li Y., Fang Q., Zhang L. (2019). Fabricated Biobased Eucommia Ulmoides Gum/Polyolefin Elastomer Thermoplastic Vulcanizates into a Shape Memory Material. Ind. Eng. Chem. Res..

[8] Qi X., Zhao X., Li Y., Zhang J., Zhang L., Yue D. (2020). A high toughness elastomer based on natural Eucommia ulmoides gum. J. Appl. Polym. Sci..

[9] Wang Y., Liu J., Xia L., Shen M., Xin Z. (2019). Super-tough poly(lactic acid) thermoplastic vulcanizates with heat triggered shape memory behaviors based on modified natural Eucommia ulmoides gum. Polym. Test..

[10] Wang Y., Xia L., Xin Z. (2018). Triple shape memory effect of foamed natural Eucommia ulmoides gum/high-density polyethylene composites. Polym. Adv. Technol..

[11] Tian M., Gao W., Hu J., Xu X., Ning N., Yu B., Zhang L. (2020). Multidirectional Triple-Shape-Memory Polymer by Tunable Cross-linking and Crystallization. ACS Appl. Mater. Interfaces.

[12] Kugimoto D., Kouda S., Yamaguchi M. (2019). Improvement of mechanical toughness of poly(lactic acid) by addition of ethylene-vinyl acetate graft copolymer. Polym. Test..

[13] Henderson A. (1993). Ethylene-vinyl acetate (EVA) graft copolymers: A general review. IEEE Electr. Insul. Mag..

[14] Qi X., Yang W., Yu L., Wang W., Lu H., Wu Y., Zhu S., Zhu Y., Liu X., Dong Y. (2019). Design of Ethylene-Vinyl Acetate graft copolymer Fiber with Two-Way Shape Memory Effect. Polymers.

[15] Huskić M., Šebenik A. (1993). Characterization of crosslinked ethylene—Vinylacetate graft copolymers. Polym. Int..

[16] Han J.-L., Lai S.-M., Chiu Y.T. (2018). Two-way multi-shape memory properties of peroxide crosslinked ethylene vinyl-acetate graft copolymer (EVA)/polycaprolactone (PCL) blends. Polym. Adv. Technol..

[17] Sun L., Lu X., Bai Q., Wang Z. (2022). Triple-shape memory materials based on cross-linked ethylene-acrylic acid graft copolymer and ethylene-vinyl acetate graft copolymer. Polym. Eng. Sci..

[18] Wang Y., Pei X., Xia L., Xin Z. (2021). Covalent crosslinks turn natural Eucommia ulmoides gum/polybutene-1 composites into multiple shape memory materials. Polym. Compos..

[19] Zhou X., He T., Jiang Y., Chang S., Yu Y., Fang X., Zhang Y. (2021). A Novel Network-Structured Compatibilizer for Improving the Interfacial Behavior of PBS/Lignin. ACS Sustain. Chem. Eng..

[20] Rodriguez J., Giraldo D., Restrepo J., Colorado H. (2020). Cross-linked ethylene-vinyl acetate-poly(urethane) temperature-memory composite actuator. J. Compos. Mater..

[21] Soares B.G., Cario F.O. (2006). Reactive compatibilization of polystyrene/poly(ethylene–co–vinyl acetate) (EVA) blends. J. Appl. Polym. Sci..

[22] Srimalanon P., Prapagdee B., Markpin T., Sombatsompop N. (2018). Effects of DCP as a free radical producer and HPQM as a biocide on the mechanical properties and antibacterial performance of in situ compatibilized PBS/PLA blends. Polym. Test..

